# Retraction Note: Cyclin D3 predicts disease-free survival in breast cancer

**DOI:** 10.1186/s12935-026-04480-3

**Published:** 2026-10-07

**Authors:** Yayun Chi, Sheng Huang, Mengying Liu, Liang Guo, Xuxia Shen, Jiong Wu

**Affiliations:** 1 Department of Breast Surgery, Breast Cancer Institute, Fudan University Shanghai Cancer Center, Building 7, No. 270 Dong An Road, Shanghai, 200032 China; 2 Department of Pathology, Fudan University Shanghai Cancer Center, Shanghai, 200032 China; 3 Department of Oncology, Shanghai Medical College, Fudan University, Shanghai, 200032 China

**Retraction Note: Cancer Cell Int (2015) 15:89**

**https://doi.org/10.1186/s12935-015-0245-6**.

The Editors-in-Chief have retracted this Article. Following publication, concerns were raised by the authors, including but not limited to the following issues:

- The Migration image in the Control (Con) group partially overlaps with the Invasion image in the same group in Fig. 3.
- The Migration image in the siCCND3 group partially overlaps with the Invasion image in the same group in Fig. 3.

The Editors-in-Chief therefore no longer have confidence in the results and conclusions presented in this Article.

The authors have not responded to any correspondence from the Editor about this retraction.

